# Facile Synthesis of Carbon-Based Inks to Develop Metal-Free ORR Electrocatalysts for Electro-Fenton Removal of Amoxicillin

**DOI:** 10.3390/gels10010053

**Published:** 2024-01-11

**Authors:** Laura Carolina Valencia-Valero, Edgar Fajardo-Puerto, Abdelhakim Elmouwahidi, Esther Bailón-García, Francisco Carrasco-Marín, Agustín Francisco Pérez-Cadenas

**Affiliations:** UGR-Carbon, Materiales Polifuncionales Basados en Carbono, Dpto. Química Inorgánica, Unidad de Excelencia de Química Aplicada a Biomedicina y Medioambiente, Universidad de Granada (UEQ-UGR), 18071 Granada, Spain; lauracarolina.valencia@urv.cat (L.C.V.-V.); aelmouwahidi@ugr.es (A.E.); estherbg@ugr.es (E.B.-G.); fmarin@ugr.es (F.C.-M.)

**Keywords:** wastewater, ORR, electro-Fenton, carbon gels, xerogels

## Abstract

The electro-Fenton process is based on the generation of hydroxyl radicals (OH•) from hydroxide peroxide (H_2_O_2_) generated in situ by an oxygen reduction reaction (ORR). Catalysts based on carbon gels have aroused the interest of researchers as ORR catalysts due to their textural, chemical and even electrical properties. In this work, we synthesized metal-free electrocatalysts based on carbon gels doped with graphene oxide, which were conformed to a working electrode. The catalysts were prepared from organic-gel-based inks using painted (brush) and screen-printed methods free of binders. These new methods of electrode preparation were compared with the conventional pasted method on graphite supports using a binder. All these materials were tested for the electro-Fenton degradation of amoxicillin using a homemade magnetite coated with carbon (Fe_3_O_4_/C) as a Fenton catalyst. All catalysts showed very good behavior, but the one prepared by ink painting (brush) was the best one. The degradation of amoxicillin was close to 90% under optimal conditions ([Fe_3_O_4_/C] = 100 mg L^−1^, −0.55 V) with the catalyst prepared using the painted method with a brush, which had 14.59 mA cm^−2^ as *J_K_* and a H_2_O_2_ electrogeneration close to 100% at the optimal voltage. These results show that carbon-gel-based electrocatalysts are not only very good at this type of application but can be adhered to graphite free of binders, thus enhancing all their catalytic properties.

## 1. Introduction

Antibiotics are pharmaceutical compounds that are widely used for different infectious disease treatments in humans and animals, generating a possible effect on human and ecological health [1,2]. The principal problem associated with antibiotics is the generation of antibiotic resistance genes (ARGs), which reduces their efficacy against human and animal pathogens, posing a risk to public health [3,4,5].

Amoxicillin (AMX) is an antibiotic of class β-lactam, which is considered safe for use even in pregnant and lactating women; however, its metabolization in the human body is low, whereby between 70 and 90% of AMX is released to the environment unchanged [6,7,8]. Due to its solubility, biodegradability, stability and polarity, AMX is not degraded completely in wastewater treatment plants; for this reason, different methods (adsorption, advanced oxidation processes, etc.) [9,10,11,12] for the removal of this antibiotic from waters have been proposed [13].

Advanced oxidation processes (AOPs) are based on the generation of hydroxyl radicals (OH•), which degrade organic components present in water solutions [14,15]. Some AOPs are as follows: photocatalysis, electrocatalysis, Fenton-like oxidation or persulfate oxidation [16]. Processes based on electrocatalysis take their name from electrochemical advanced oxidation processes (EAOPs), where generally no type of external chemical is required to treat contaminants in water solutions [17]. The electro-Fenton process (EF) is one of the most popular EAOPs, showing high mineralization levels due to the direct generation of hydrogen peroxide (H_2_O_2_) at the cathode by the reduction reaction of oxygen (ORR) through a two-electron path (2e^−^) (Equation (1)) and the formation of OH• radicals from the generated H_2_O_2_ by the Fenton catalyst (Equation (2)) [18,19,20].

O_2_ + 2H^+^ + 2e^−^ → H_2_O_2_ (1)

Fe^2+^ + H_2_O_2_ + H^+^ → Fe^3+^ + H_2_O + OH• (2)

Fe^3+^ + e^−^ → Fe^2+^ (3)

Pollutants + OH• → Intermediates(4)

Intermediates + OH• → H_2_O + CO_2_ (5)

The ORR catalyst has a key role in the EF process and, for this reason, it is crucial for the development of catalysts with high selectivity for H_2_O_2_. Traditionally, ORR catalysts based on Pt and Pd are considered the best candidates; however, their high cost makes the use of these in the EF process difficult [21]. Carbon-based materials have been evaluated as ORR catalysts due to their low cost and high selectivity for H_2_O_2_ [22]. Qin et al. [23] synthesized hollow porous carbon spheres with high activity ORR 2e^−^ (higher than that reported in more advanced Fenton catalysts), which was attributed to the presence of sp^3^ carbon and sp^2^ defects. Garza-Campos et al. [24] developed a cathode with mesoporous carbon obtained using a soft template with a current efficiency closer to 100%, attributed to ORR 2e^−^ predominance. Pishnamaz et al. [25] modified nickel foam with single-wall and multi-wall carbon nanotubes (SWCNTs and MWCNTs, respectively) and found that SWCNTs produced more H_2_O_2_ than MWCNTs, due principally to their higher surface area. Graphene is another new carbon material studied as a metal-free ORR catalyst. Wang et al. [26] fabricated an electrode of graphene aerogel and calculated the electron transfer number between 1 and 2 in the range of the selected potential. Wang et al. [27] made a cathode of reduced graphene-oxide-grafted carbon fiber and determined that the modified electrode had a 45% higher H_2_O_2_ accumulation than the material without graphene. More specifically, carbon gels have recently and successfully been applied into an electro-Fenton process for antibiotic elimination from water [28], or simple ORR [29], and CO_2_ electro-transformation [30] due to their unique textural and chemical characteristics.

The use of polytetrafluoroethylene (PTFE) emulsions as a binder in the preparation of electrodes based on carbon materials is very common [31,32]; however, it is possible that PTFE generates jams and blockages in pores, affecting the electrocatalytic yield. In this work, metal-free electrocatalysts based on carbon xerogel/graphene oxide (OG) were synthesized and used as ORR electrocatalysts in the degradation of amoxicillin (AMX) using an electro-Fenton process, which also involved the synthesis and use of a homemade carbon/magnetite catalyst as a heterogeneous Fenton catalyst. The ORR electrocatalysts were made from the development of a carbon/OG ink, which was deposited on the graphite cathode using two different painting methods, brush and screen-printed, as well as the preparation of one ORR electrocatalyst using the same ink, PTFE, using a conventional method for comparison.

## 2. Results and Discussion

### 2.1. Catalysts ORR

#### 2.1.1. Porosity and Surface Area Determination

**Figure 1:**
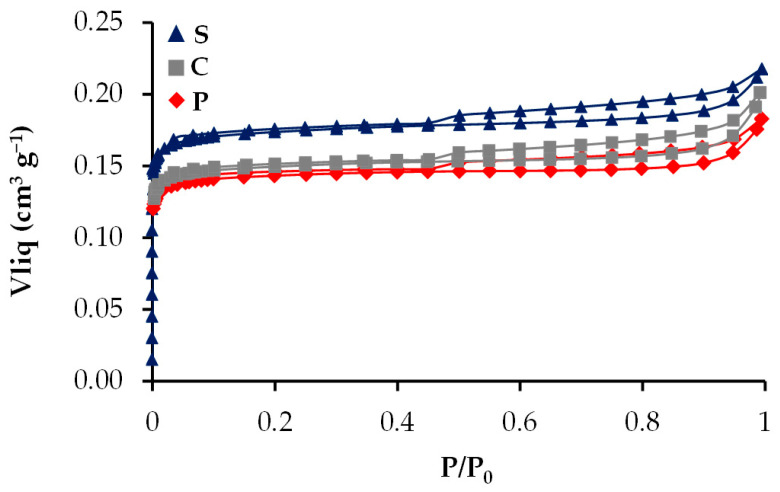
N_2_ adsorption–desorption isotherms at 77 K.

The N_2_ adsorption–desorption isotherms of the samples prepared by painting by brush (P), screen printing (S) and the conventional method (C) are shown in Figure 1. All samples show a mix of isotherms type I and IV with hysteresis loop type 4, which is typical for mesoporous materials [33]. The difference among the samples could be attributed to the dispersion type effected in each method; due to the printed screen, the ink is subjected to a pressure difference, which can result in a better dispersion of the particles. The surface area values and the volume of micro (W) and mesopores are shown in Table 1, where it is evident that there are no significant differences among the samples, although sample S shows all the highest values and C the lowest mesopore volume.

#### 2.1.2. Raman Characterization

**Figure 2:**
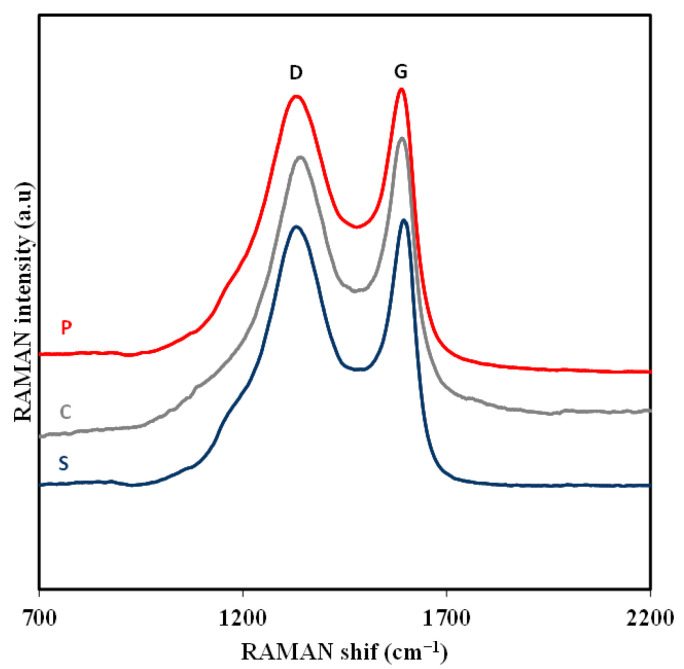
Raman spectra of all samples.

The Raman spectra of the samples P, C and S are shown in Figure 2 and were used to determine the degree of graphitization. All samples show the band associated to the vibrational modes of the graphite structure (G at 1590 cm^−1^) and the graphite structure defects (D 1340 cm^−1^) [34,35]. The intensity ratio I_D_/I_G_ is used typically as an indicator of the defect density level [36]. The ratio I_D_/I_G_ has values of 0.988, 0.976 and 0.982 for S, C and P, respectively, indicating that the samples have a good graphitization grade and that there is no significant difference among them.

#### 2.1.3. XPS Characterization

**Figure 3:**
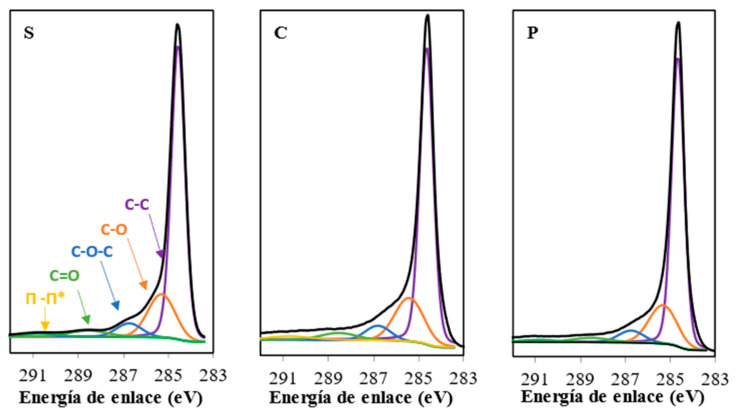
XPS spectra of C_1S_ regions.

The chemical composition of the surface was studied by XPS, and the C_1S_ and O_1S_ regions were analyzed in all the samples. All spectra are very similar among the samples. Figure 3 shows the three C_1S_ spectra as an example. The deconvolution results and the binding energy peaks are collected in Table 2.

We use as a reference the 285.5 eV value to calibrate the C_1s_ spectra, and the peaks 284.6, 285.4, 286.8, 288.5 and 290.7 were attributed to C-C, C-O, C-O-C, C=O and CO_2_, respectively [37,38,39,40,41].

The more significative difference among the samples is the content of oxygen, which is slightly higher in sample C. Therefore, the chemical composition of the samples can be considered practically equal.

#### 2.1.4. Morphology

**Figure 4:**
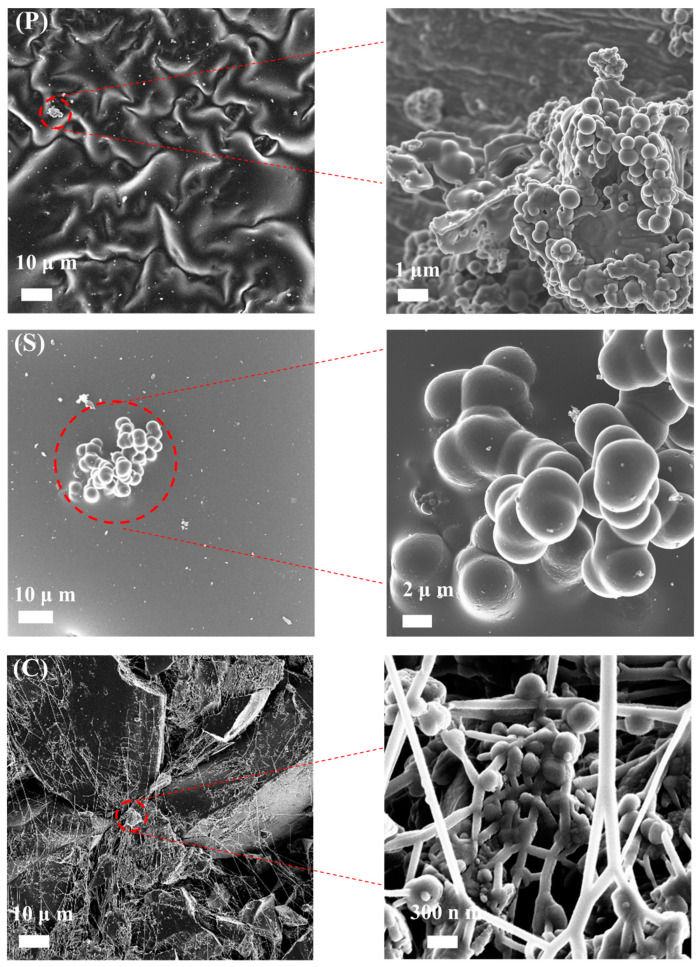
SEM images.

The morphology of the samples was examined by SEM (see Figure 4), and it was possible to observe significant differences among the samples, mainly in roughness. The sample with the greatest roughness was P, which shows several possible channels or slits that could facilitate access to more active sites.

Sample S is the smoothest and shows fewer microspheres exposed on the surface; meanwhile, in the C sample, it is possible to observe the binder that generates a thread that traps the carbon spheres, generating more agglomerations.

**Figure 5:**
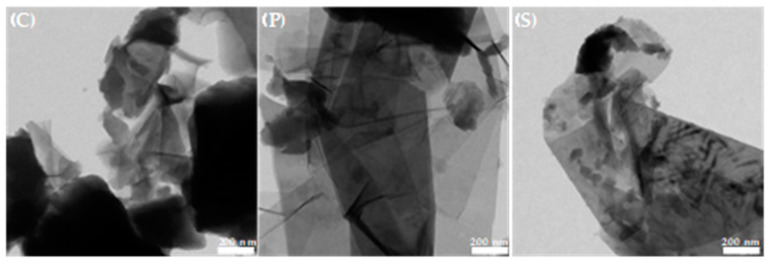
TEM images.

The morphology of the samples was examined by TEM (Figure 5), where the OG structure can be observed with the typical texture (wrinkled sheets) and a good exfoliation of these [42,43]. At this level of magnification, all three samples look relatively similar.

#### 2.1.5. Electrochemical Characterization

**Figure 6:**
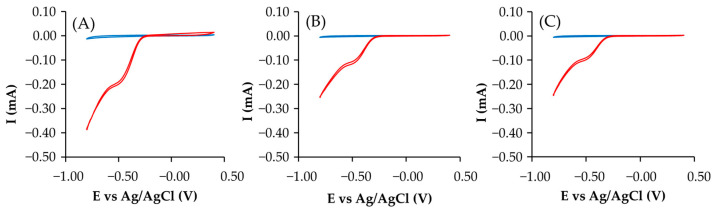
CV (**A**) sample C, (**B**) sample P and (**C**) sample S; O_2_ (red) and N_2_ (blue).

Figure 6 shows the CV in the presence and absence of O_2_, with the peak characteristics of the oxygen reduction showing activity as an ORR catalyst.

**Figure 7:**
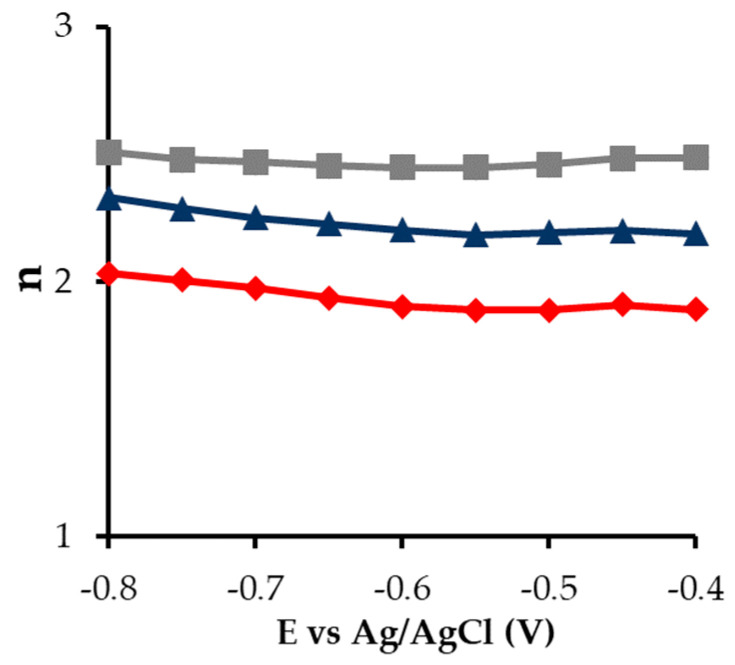
Number of transferred electrons 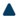 S, 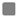 C and 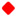 P.

Figure 7 shows the number of electrons transferred in the reaction obtained with RRDE. All samples have a preferential selectivity to a two-electron path throughout the voltage range, especially sample P, and without apparent mechanic changes in the potential range studied. Some authors have attributed an improvement of the conductivity to better electrochemical activities [44,45]; however, the Raman spectra do not show significant differences among the samples.

Table 3 shows the parameters of the Koutecky–Levich equation, from which it is possible to obtain information about the reaction mechanism of these electrocatalysts [46,47]. In this context, all samples work close to a two-electron pathway mechanism.

On the other hand, the initial potential is more positive in sample C, followed by P and S, which is related to better electron transfer ability [48]. The diffusion kinetic current density (*J_K_*) shows much higher values for the samples S and P (14.35 and 14.59, respectively), which can be due to a combination of factors like lower oxygen content and a much higher mesoporosity, which could be a direct consequence of the new preparation method versus the conventional one. The direct positive relation between current density and ORR performance is very well known [49]. In terms of electrocatalytic results, sample P is slightly superior to sample S (higher *J_k_* and n values and lower initial potential). Therefore, among these catalysts, sample P brings together the best combination of textural and electrochemical characteristics, such as higher *J_k_* and mesopore volume, also showing a quite adequate electron transfer value. It is also important to highlight the type of morphology caused by the painting method in terms of microroughness that can improve the accessibility of the mesoporosity in particular, and of the active sites in general to the electrolyte.

**Figure 8:**
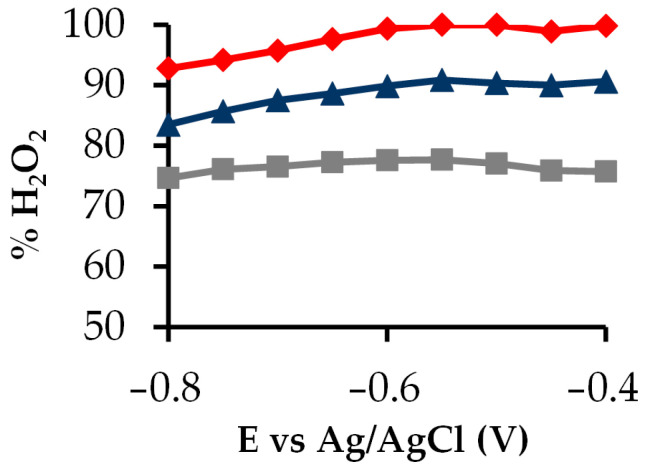
H_2_O_2_ electrogeneration at each potential with 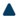 S, 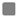 C and 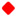 P.

Figure 8 shows the electrogeneration of H_2_O_2_ at each potential for the samples S, C and P. For all samples, the H_2_O_2_ production is constant in all potential ranges, indicating that there are no changes of selectivity, maintaining a good stability at each potential. From these results, it is considered that the H_2_O_2_ production is heavily dependent on a good balance between the textural and electrochemical characteristic; the lowest *J_k_* and mesopore volume of sample C could justify its penalty in the H_2_O_2_ generation sequence.

### 2.2. Catalyst Fenton

#### 2.2.1. X-ray Diffraction

**Figure 9:**
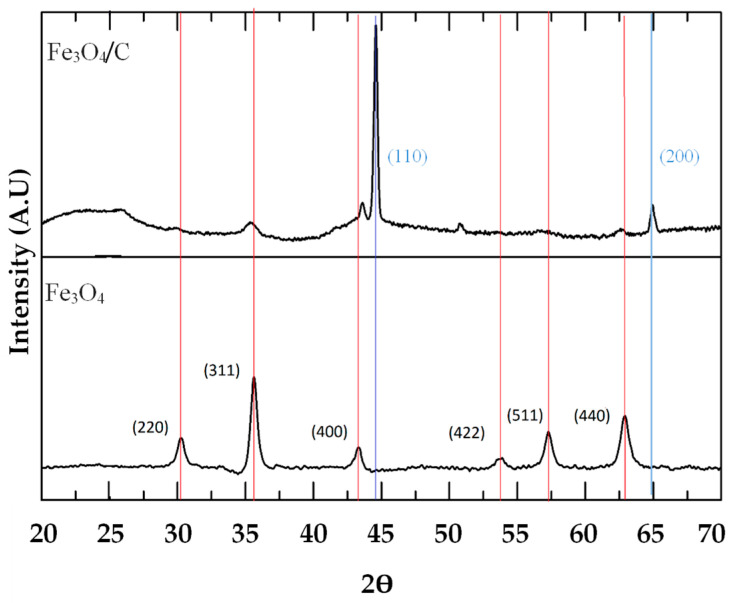
XRD patterns of Fe_3_O_4_/C and Fe_3_O_4_.

The XRD spectra of the Fenton catalyst can be observed in Figure 9, where it is possible to observe a peak characteristic of the crystalline planes (220), (311), (400), (422), (511) and (440), typical of magnetite in the sample Fe_3_O_4_ [50,51], indicating that the synthesis was made successfully.

The peak (311) was used to determine the crystallite size by Scherrer´s equation, giving a mean diameter of 12.9 nm as the result. The sample Fe_3_O_4_/C shows the peaks associated to the crystalline planes (110) and (200) of zero-valent iron (or pure Fe phase); the other peaks can easily be mistaken with magnetite peaks in small intensity [52,53]. These results indicate that the carbonization to 900 °C principally occasioned the magnetite passing to zero-valent iron.

#### 2.2.2. Morphology

**Figure 10:**
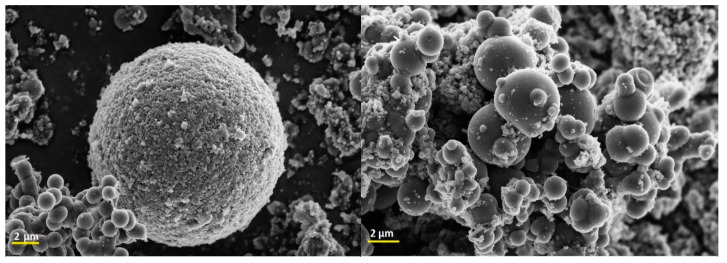
SEM images of Fe_3_O_4_/C.

Figure 10 shows SEM images of the homemade Fe_3_O_4_/C catalyst. It is formed by microspheres of different sizes, some of them partially fused.

### 2.3. Electro-Fenton Experiments (EF)

The optimal conditions for the EF process were selected by a previous test of amoxicillin (AMX) degradation, which were evaluated: the Fenton catalyst concentration, the cathodic potential and obviously the effect of the deposition method on the electrocatalyst preparation.

**Figure 11:**
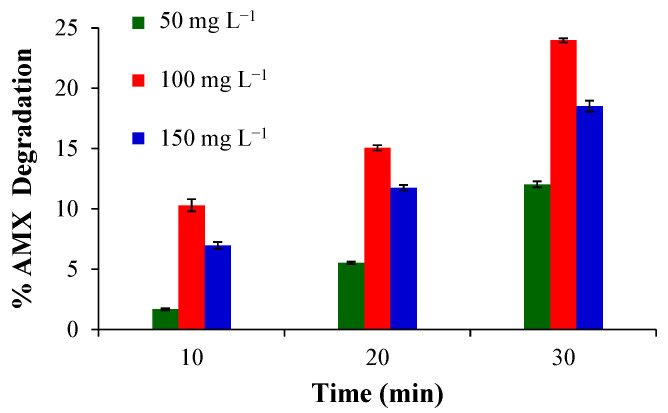
AMX degradation obtained using different concentrations of sample P.

Figure 11 shows the Fenton catalyst concentration effect, using sample P as the ORR catalyst. The degradation is better when the concentration increases from 50 to 100 mg L^−1^, due principally to the fact that more Fe^2+^ in the solution can generate more •OH (Equation (2)). However, when the concentration is higher (150 mg L^−1^), there are no significative improvements; this is due to some factors like the agglomeration between particles that can decrease the rate of OH desorption, which is one of the three key steps in the generation of hydroxyl radicals (adsorption, homolysis and desorption) and excessive accumulation of iron that finally eliminates the •OH [54,55,56]. Based on these results, the concentration of 100 mg L^−1^ was selected as the most adequate for our experimental system.

**Figure 12:**
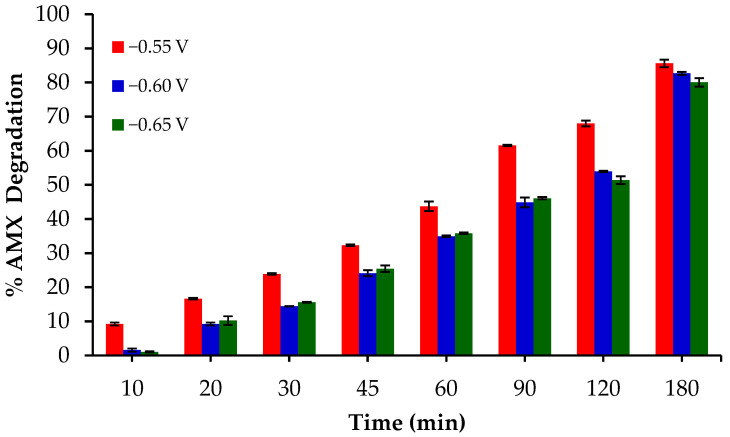
AMX degradation obtained at different potentials with the catalyst P.

On the other hand, three cathodic potentials were tested in the range between −0.55 and −0.65 V (Figure 12), determining that the best potential was −0.55 V, as the highest percentage of degradation in our experimental conditions was obtained at this value.

The degradation of AMX between the voltages −0.60 and −0.65 does not show significant differences, which could be due to a competence with the hydrogen evolution reaction (HER) or even an ORR 4e^−^ [57,58], decreasing the efficiency of the EF process. Besides, the use of lower potentials is always better from an economical point of view.

**Figure 13:**
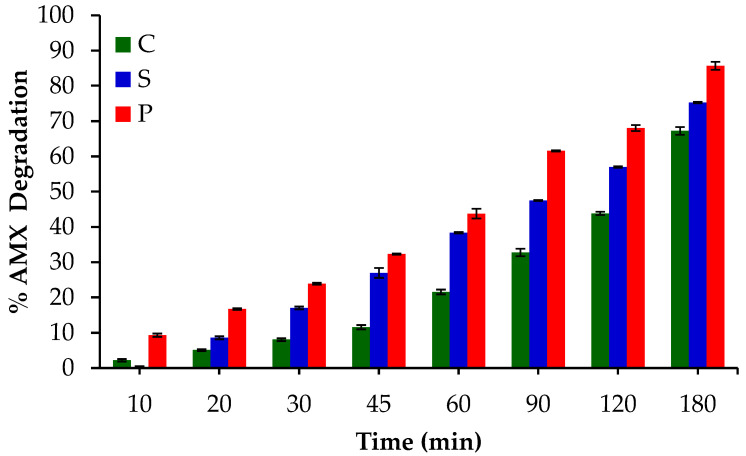
AMX degradation obtained with the catalysts prepared through the three different methods.

Finally, Figure 13 shows the deposition method effect in the degradation of AMX by EF. Over the entire reaction time range, sample P shows the highest percentage of AMX degradation, with samples P and S being better for this application than sample C, which shows the success of these new methods of preparation or deposition of electrocatalysts.

These results of AMX degradation are directly related to the ORR performance and H_2_O_2_ electrogeneration already discussed in Section 2.1.5. The painted method (sample P) produces a non-uniform micro-deposition generating micro-roughness, which leaves ORR active sites more exposed to the solution, improving the final AMX degradation. Additionally, the conventional method (sample C) is also limited by the use of a binder, which probably makes the electrocatalytic process less efficient. In a similar way, the screen-printed process (sample S), due to the excellent uniformity of the deposited layers, can limit the solution accessibility in comparative terms with sample P.

**Figure 14:**
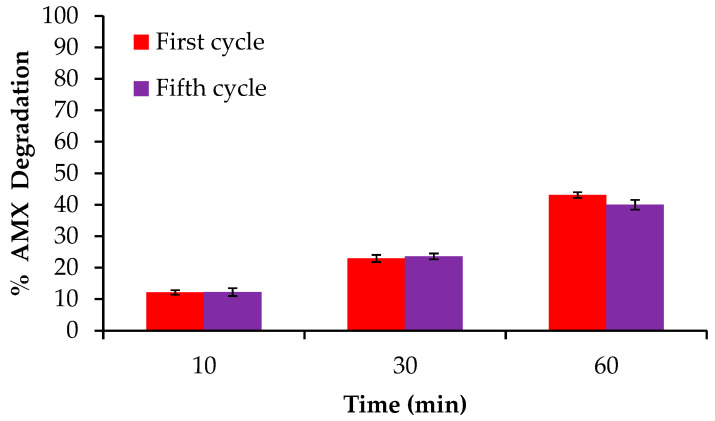
Reusability study carried out with the sample P.

Finally, a minimum study of reusability was carried out with sample P, as this is a critical factor to determine a possible broader application. Therefore, the same prepared electrode was tested in the optimal EF conditions by five consecutive cycles. Figure 14 shows the AMX degradation kinetic after the first and fifth cycle of use, maintaining the EF system at the same degradation capacity after 60 min.

## 3. Conclusions

The results obtained in this work show the successful synthesis of ORR catalysts based in carbon xerogels doped with graphene oxide, which could be confirmed by different methods, with a very good performance to be coupled in electro-Fenton systems for amoxicillin degradation. In comparative terms, the conventional ORR electrocatalysts preparation method, consisting of a previous carbonization of the carbon precursor and its later deposition on the cathode using binders (sample C), can be broadly improved by the development of the carbon-gel/OG-based ink discussed here. This ink can be deposited on the cathode using a brush (sample P) or an airbrush (sample S) and later carbonized.

In this way, sample P was the one that showed the best electrochemical behavior; consequently, the best result was obtained in the degradation of AMX (close to 90%) after its coupling to the EF system. The explanation of this result must be understood as a combination of different factors: the unique microroughness generated in the cathode together with the high volume of mesopores obtained provides a better set of ORR parameters (*J_k_* 14.59 mA cm^−2^, 2.03 transferred electrons and initial potential of −0.25 V), producing the greatest amount of H_2_O_2_ among the samples, which is essential for efficient electro-Fenton degradation of AMX.

## 4. Materials and Methods

### 4.1. Synthesis of Materials

#### 4.1.1. Graphene Oxide (OG)

The OG was synthetized by a modified Hummers method [59], mixing graphite (4 g) with sulfuric acid (H_2_SO_4_) (100 mL), added drop by drop with agitation in an ice bath. Then, potassium permanganate (KMnO_4_) (12 g) was added and the resulting mix was kept under constant agitation for 2 h at 35 °C. The obtained mix was diluted with water (200 mL); subsequently, H_2_O_2_ (30% *w v*^−1^) was added until a yellow solution formed. Then, the material was purified with hydrochloric acid and washed several times with water until reaching neutral pH. Finally, the material was dried at 60 °C in 24 h.

The obtained solid was dispersed in distilled water (200 mL) and sonicated for 1 h; subsequently, the dispersion was centrifugated (20 min to 3000 rpm) to eliminate the oxide graphite particles. With the final solution, a suspension of OG (0.8 g L^−1^) was prepared.

#### 4.1.2. Magnetite (Fe_3_O_4_)

The magnetite was synthesized by co-precipitation chemical method [60], mixing 50 mL of ferrous chloride with 100 mL of ferric chloride (0.5 M). Then, 75 mL of ammonium hydroxide (25%) was added drop by drop, and the solution was maintained with constant agitation for 10 min. Finally, the obtained solid was separated and washed several times with water and ethanol. The final material was dried for 8 h at 60 °C.

The coating with carbon was made following a typical sol–gel method [61], using resorcinol–formaldehyde as carbon precursors and 3% of magnetite. The procedure was carried out with 11 mL of Span 80 (S) dissolved in 250 mL of n-heptane and heated at 70 °C with reflux and mechanical stirring (450 rpm) for 1 h. Then, the magnetite in solution (1.05 g in 25 mL of NH_4_OH (30%)) was added and maintained for 2 h. Another solution was prepared with 22.5 g of resorcinol (R), 12.5 mL of formaldehyde (F) and 29 mL of water (W), which was added dropwise to the solution of n-heptane. The obtained gel was aged at 70 °C for 24 h under stirring. Then, the suspension was filtered and the solid was placed in acetone for 5 days (changing acetone twice daily). The obtained material was dried by infrared light and, finally, carbonized at 900 °C for 2 h with N_2_ flow at 300 mL min^−1^ and a heating rate of 2 °C min^−1^.

#### 4.1.3. Preparation of the Xerogel/OG Ink

The xerogel was synthetized from resorcinol (R) (5 g) and formaldehyde (F) (7.35 g), and dissolved in water (OG suspension) (W). Then, 2-hydroxypidine (0.088 g) was added as catalyst (Z). The mixture was heated in a microwave at 400 W power, in an argon atmosphere, for two batches of 5 min, waiting between both batches for the ink to cool to room temperature. In all cases, the used molar relations were R/F 0.5, W/R 50 and R/Z 500.

### 4.2. Deposition Methods

The xerogel/OG ink was deposited by different methods on graphite sheets (7 cm × 1 cm) used as support.

#### 4.2.1. Conventional Catalyst (Sample C)

For the conventional preparation of the electrode, the ink was previously dried in an oven at 100 °C for 12 h. Subsequently, it was carbonized in argon with a heating rate of 3 °C min^−1^ up to 850 °C, maintaining this temperature for 1 h. The obtained carbon xerogel was grounded to a fine powder with which a paste of 150 mg of xerogel and polytetrafluoroethylene (PTFE) (60% suspension in water) was made, mixed homogeneously at a proportion of 90:10. This mixture was homogeneously pasted by mechanical method on both sides of the graphite sheet and dried at 80 °C.

#### 4.2.2. Painted Catalyst (Sample P)

The electrode was prepared using a brush to deposit the xerogel/OG ink in each support face until obtaining a constant mass of 0.5 g. After each deposition, the electrode was placed under infrared light for 1 min. Finally, it was carbonized in argon with a heating rate of 3 °C min^−1^ up to 850 °C, maintaining this temperature for 1 h.

#### 4.2.3. Screen-Printed Catalyst (Sample S)

This method was like the painted method, changing the brush to an airbrush for the ink deposition. The other conditions were equal.

### 4.3. Characterization

#### 4.3.1. Chemical and Textural Characterization

The Brunauer–Emmett–Teller (B.E.T), Dubinin–Radushkevic (DR) and Density Functional Theory (DFT) methods were applied to the data obtained from the N_2_ adsorption isotherms at 77 K to obtain the apparent surface area and micropore parameters. DR and DFT were used to calculate the total micropore volume (W_0_/W_DFT_) and the mean micropore width (L_0_/L_DFT_). The total pore volume was considered as the N_2_ volume adsorbed at a relative pressure of 0.95 (V_0.95_). Furthermore, the mesopore volume (V_meso_) was obtained from the difference between V_0.95_ and W_0_.

RAMAN spectroscopy was performed with a Micro-Raman spectrometer (JASCO NRS-5100, laser DXR 532 nm) in a range between 200 and 3000 cm^−1^, and at 24 Mw.

The surface composition was obtained by X-ray photoelectron spectroscopy using an ESCA 5701 Physical Electronics (PHI) system (equipped with MgKa anode, model PHI 04-548; X-ray source (hγ = 1253.6 eV)) and hemispherical electron. For the analysis of the XPS peaks, the C_1S_ peak position was at 284.6 eV.

The samples’ morphology was observed by scanning electron microscope (SEM) and transmission electron microscopy (TEM) in the microscopies AURIGA (FIB-FESEM) and LIBRA 120 Plus, respectively, at the “Centro de Instrumentación Cientifica de la Universidad de Granada.

#### 4.3.2. Electrochemical Characterization

The electrochemical characterization was carried out in a cell of 3 electrodes controlled by a potentiostat multichannel Biologic VMP, using Ag/AgCl, Pt and a rotating ring–disk electrode (RRDE) as the reference electrode, counter electrode and working electrode, respectively.

The RRDE was prepared with 10 μL of a solution of 5 mg of material in 1 mL of a mix of Nafion (5%) and water (relation 1:9).

The cyclic voltametric (CV) experiments were carried out in N_2_ or O_2_ in a potential range of −0.75 V to −0.45 V, with a scan speed of 5 mVs^−1^ and 50 mVs^−1^. The linear sweep voltammetry (LSV) was carried out in O_2_ at different rotation speeds (500, 1000, 1500, 2000, 2500, 3000 and 4000 rpm) with exploration speed of 5 mVs^−1^ in the same potential range of CV. In the two cases, the support electrolyte used was KOH (0.1 M). To calculate the number of transfer electrons, the data obtained from LSV experiments were adjusted to the Koutecky–Levich model (Equation (1)) [62].

(6) $$\frac{1}{J}=\frac{1}{J_{K}}+\frac{1}{0.2\;nF{(D}_{o}^{2/3})v^{-1/6}C_{o}w^{1/2}}$$

where *J* is current density, *J_K_* is current density kinetic, *ω* is rotation speed, *F* is the Faraday constant, *D_o_* (1.9 × 10^−5^ cm^2^ s^−1^) is oxygen coefficient diffusion, *υ* (0.01 cm^2^ s^−1^) is viscosity and *C_o_* is the oxygen concentration (1.2 × 10^−6^ mol cm^−3^).

### 4.4. Electro-Fenton Processes

The electro-Fenton process was carried out using a standard three-electrode electrochemical cell with capacity for 0.125 L of solution at room temperature. The AMX solution was prepared with a dilution of Na_2_SO_4_ (0.5 M) with continuous agitation. The potentiostat was maintained in potentiostatic mode at different evaluated values (−0.55, −0.60 and −0.65 V). The reference electrode was Ag/AgCl, and the counter electrode used was platinum wire. The pH was 6.0 (natural pH solution). As the Fenton catalyst, magnetite coated with carbon (Fe_3_O_4_/C) was used. AMX concentrations in solution were determined by a UV–vis spectrophotometer at 229 nm. As a general protocol, each type of electro-Fenton test was repeated three times with a new sample of each specific catalyst.

## Figures and Tables

**Table 1:** Textural characteristics of all samples obtained by N_2_ adsorption at 77 K.

Sample	S_B.E.T_	S_DFT_	S_DR_	W_0_	L_0_	W_DFT_	L_DFT_	V_0.95_	V_meso_
	m^2^ g^−1^	m^2^ g^−1^	m^2^ g^−1^	cm^3^ g^−1^	nm	cm^3^ g^−1^	nm	cm^3^ g^−1^	cm^3^ g^−1^
S	436	506	492.2	0.170	0.63	0.19	0.61	0.218	0.043
C	383	448	424.2	0.150	0.60	0.17	0.61	0.171	0.020
P	363	428	408.6	0.145	0.65	0.15	0.61	0.183	0.038

**Table 2:** Characterization data obtained from XPS in C_1S_ region.

Bond	Peak	S (%)	C (%)	P (%)
C-C	284.6	70.3	68.8	71.2
C-O	285.4	18.7	19.7	18.9
C-O-C	286.8	5.5	5.7	5.3
C=O	288.5	3.3	3.6	2.7
CO_2_	290.7	2.1	2.2	1.9
Total C	---	93.8	93.0	94.1
Total O_2_	---	6.2	7.0	5.9

**Table 3:** Parameters of Koutecky–Levich obtained at −0.8 V, and initial potential.

Sample	*J_K_* _mA cm_^−2^	n	E° Initial V
S	14.35	2.33	−0.25
C	9.00	2.50	−0.23
P	14.59	2.03	−0.25

## Data Availability

The data presented in this study are openly available in article.
